# Bond strength and interfacial stability of a universal adhesive to alendronate-treated dentin

**DOI:** 10.1038/s41598-026-41664-3

**Published:** 2026-03-24

**Authors:** Hala S. Salem, Enas T. Enan, Hamdi Hamama, Marwa S. Ismail

**Affiliations:** 1 Dental Biomaterials Department, Faculty of Dentistry, Mansoura University, Mansoura, Egypt; 2 Conservative Dentistry Department, Faculty of Dentistry, Mansoura University, Mansoura, Egypt; 3 Faculty of Oral and Dental Medicine, Alsalam University, Tanta, Egypt

**Keywords:** Alendronate, Chlorohexidine, Dentin bonding agents, Microtensile bond strength, Nanoleakage, Diseases, Health care, Materials science, Medical research

## Abstract

The enzymatic degradation of the hybrid layer by endogenous matrix metalloproteinases (MMPs) represents the major cause of resin–dentin bond strength deterioration. The current study assessed the influence of dentin pre-bonding treatment with alendronate, an MMP inhibitor, on the efficacy of a universal adhesive.Ninety-six extracted molars were randomly allocated into four groups (*n* = 24) based on dentin surface treatment: no treatment (control), 2 wt% chlorhexidine, 0.03 wt% alendronate, and 0.3 wt% alendronate. Each group was further classified into two sub-divisions (*n* = 12) according to the adhesive application mode: etch-and-rinse (ER) and self-etch (SE). On flat dentin surfaces, the treatment solution was applied, followed by application of universal adhesive (All Bond UNIVERSAL, BISCO) and composite buildup. Eighty teeth were used for the assessment of microtensile bond strength (µTBS), failure mode, and nanoleakage, while the remaining sixteen teeth were examined using scanning electron microscopy (SEM) to assess the bonded interface. All tests were conducted at 2 time intervals; after 24 h and after aging for 5000 thermocycles. Statistical analysis of the µTBS data was performed using three-way ANOVA followed by Tukey’s post hoc test. The 0.3 wt% alendronate-treated ER group exhibited the highest µTBS values under both immediate and aged conditions (p value < 0.05). Using SE adhesive mode, the same treatment showed relatively higher µTBS values; however, the variations among SE groups were not significant. Failure mode analysis revealed that immediate alendronate-treated ER groups predominantly exhibited cohesive failures, whereas the ER control group presented a higher frequency of mixed failures. Following thermocycling, alendronate-treated ER groups showed a shift toward mixed failure mode. Nanoleakage analysis indicated greater silver uptake in the ER groups compared to SE groups, particularly after aging. Micromorphological surface analysis by SEM showed that the 0.3 wt% alendronate-treated ER group exhibited the most pronounced resin penetration. It has been concluded that dentin treatment with alendronate specifically 0.3 wt%, before bonding, appeared to enhance bond strength, particularly with etch-and-rinse mode. The results of failure mode analysis, nanoleakage evaluation and micromorphological interface observations confirmed these findings.

## Background

Universal adhesives are widely used because they can be applied in ER or SE modes and bond effectively to various substrates. Their clinical success is largely attributed to the 10-MDP monomer, which forms a strong bond with hydroxyapatite. This bond is exceptionally stable, characterized by the limited solubility of its calcium salt in aqueous solutions. Despite these advantages, the long-term performance of universal adhesives remains a challenge. Previous studies have shown a marked decrease in bond strength and an increase in dentin nanoleakage with aging^1,2^. The main cause of bonding drop is incomplete infiltration of resin, which leaves collagen fibrils partially or completely exposed within the hybrid layer. Exposed collagen fibrils are susceptible to both hydrolytic and enzymatic degradation, resulting in hybrid layer breakdown and a gradual decline in bond strength^3,4^.

Enzymatic breakdown of the hybrid layer, primarily caused by matrix metalloproteinases (MMPs), is a major factor contributing to failure of adhesive restorations. MMPs are endogenous Zn²⁺- and Ca²⁺-dependent proteolytic enzymes produced during dentinogenesis^5^. In human dentin, there are at least six different types of MMPs, including stromelysin–1 (MMP–3), collagenase (MMP–8), gelatinases (MMP–2 and MMP–9), membrane-type 1 (MMP–14), and enamelysin (MMP–20)^6^. Although MMPs remain in a latent form within the dentin extracellular matrix, studies have demonstrated that the acidic environment produced by adhesive systems can activate these MMPs, triggering a cascade of collagen degradation^7–10^.

Chlorhexidine digluconate (CHX) is commonly considered the gold standard MMP inhibitor for preserving hybrid layer durability. Typically, a 2.0 wt% chlorhexidine digluconate solution is applied topically after acid etching and before adhesive application. Despite its inhibitory effect, the long-term anti-proteolytic efficacy of CHX is limited by its slow leaching out of the hybrid layer due to its high molecular size and water solubility^9,11^. Therefore, finding alternatives with long-term stability is valuable^12^.

Studies have shown that alendronate, a first-line treatment for osteoporosis, can inhibit MMPs at therapeutically concentrations (0.03wt%, 0.3wt%)^13,14.^ This inhibitory action is attributed to its ability to bind to metal ions like calcium and zinc, which are very important for MMP activity. Beyond its inhibitory effect, alendronate has a unique chemical structure that explains its strong affinity for mineralized tissue^15^. It contains two phosphonate groups and a hydroxy group, which enhance the bonding to calcium. This stable bond allows alendronate to remain tightly bound to hydroxyapatite, guaranteeing its stability within dentin and suggesting a long-term anti-proteolytic effect^16^.

The current study aimed to assess the effect of pre-bond dentin surface treatment with alendronate solution on bond strength and durability of a universal adhesive. The null hypothesis assumed that pre-bonding surface treatment with alendronate would not affect the dentin bonding performance of the universal adhesive, regardless of the etching approach or aging protocol.

## Materials and methods

The present study was conducted after ethical approval from the Ethical Committee of the Faculty of Dentistry, Mansoura University (Approval No. A0202024DM).

### Sample size calculation

The required sample size was estimated using G^*^ Power (version 3.1.9.4) with an effect size set at 1.40, employing a two-tailed test, a 0.05 alpha level, and a statistical power of 80%, based on data obtained from a previous study^18^.

## Sodium alendronate (AL) solution preparation

To prepare a 0.03 wt% AL solution, 0.006 g of sodium alendronate trihydrate powder (Thermo Fisher Kandel GmbH, Kandel, Germany) was precisely weighed using an analytical balance and gradually dissolved in 20 mL of distilled water, with continuous magnetic stirring at 500 rpm for 15 min at room temperature to ensure complete homogeneity. A 0.3 wt% AL solution was prepared using 0.06 g of sodium alendronate trihydrate dissolved in 20 mL of distilled water, following the same procedure^19^.

## Teeth selection and grouping

Ninety-six sound freshly extracted human third molars were collected from the clinic of Oral Surgery Department at the Faculty of Dentistry, Mansoura University. The gathered teeth were extracted due to periodontal problems. The teeth were thoroughly cleansed with an ultrasonic scaler and polished with fine oil and fluoride-free pumice slurry to remove any debris. For disinfection, teeth were soaked in 0.5% chloramine-T for 24 h. Afterwards, a stereomicroscope (Leica, Hanau, Germany) at 40× magnification was used to evaluate the teeth, and any specimens showing enamel cracks, discoloration, or caries were excluded. Teeth were then immersed in 0.1% thymol at 4 ± 1 °C until testing^20–22^.

**Fig. 1:**
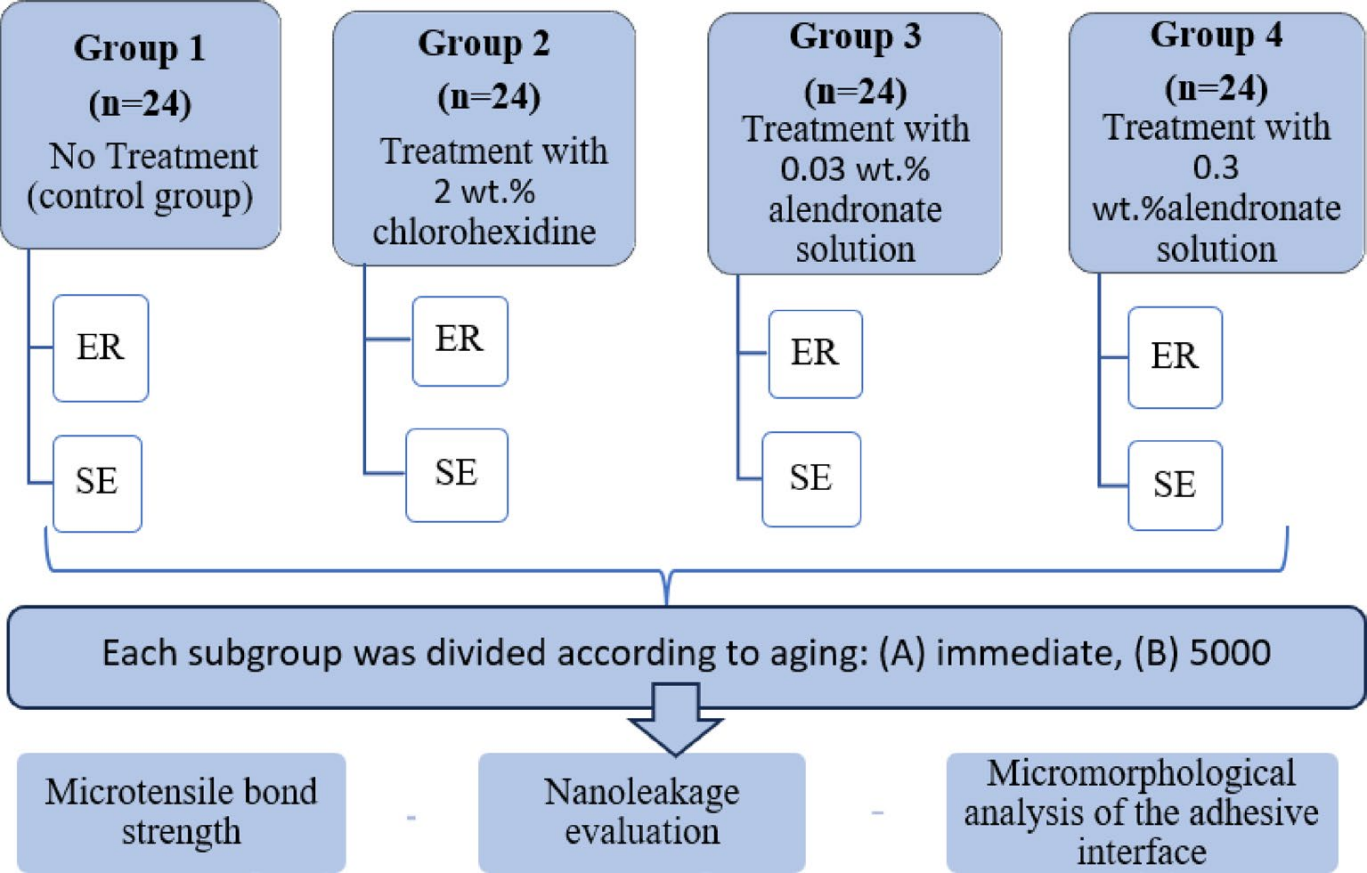
The experimental design of the study.

NBBThe teeth were then randomly allocated into four equal groups (*n* = 24) based on the dentin pre-treatment protocol: Group 1: no treatment (control), Group 2: treatment with 2 wt% chlorohexidine (CHX) solution (Free Trade Egypt, origin: India), Group 3: treatment with 0.03 wt% alendronate (AL) solution (prepared) and Group 4: treatment with 0.3 wt% alendronate (AL) solution (prepared). Then, each group was divided into two subgroups (*n* = 12) based on the adhesive technique used: etch-and-rinse (ER) or self-etch (SE). The experimental design of the study is shown in Fig. 1.

## Teeth preparation

The teeth were segmented perpendicular to their vertical axis using a low-speed, water-cooled diamond saw (Isomet, Buehler, Lake Bluff, IL, USA) to create flat mid-coronal dentin surfaces. A standardized smear layer was produced by applying the silicon-carbide abrasive paper (600 grit) in circular motions for 60 s with a low-speed straight handpiece (EX-203 C Set, NSK, Japan). For the ER specimens, dentin was etched for 30 s using phosphoric acid 37% concentration (Meta Etchant, Meta Biomed Co., Ltd., Cheongju, Korea), rinsed thoroughly, and excess water was removed using absorbent papers^17,23^.

A 20 µL volume of each treatment solution was rubbed onto the dentin surface for 60 s using a disposable microbrush. The dentin surface was then gently blotted with absorbent paper. The adhesive was applied according to the manufacturer’s protocol and polymerized for 10 s with an LED curing device (Pen Eˏ Eighteeth Medical Technology Co., Ltd., China). Composite resin (Filtek Z250, 3 M ESPE, St. Paul, MN, USA) was utilized in increments of 2-mm thickness, and each increment was light-cured for 20 s, resulting in a 4-mm buildup. All bonded specimens were then immersed in distilled water at 37 °C for 24 h to ensure complete curing^17^.

## Testing

Teeth within each subgroup were evenly divided according to the aging protocol (*n* = 6): (A) immediate (no aging), (B) 5000 thermocycles, equivalent to approximately six months of clinical service. Thermocycling was performed with a thermocycling device (Model 1100, SD Mechatronik, Feldkirchen-Westerham, Germany). Samples were alternately placed in water baths at 5 °C and 55 °C (± 2 °C). The duration of each bath was 20 s, accompanied by a transfer time of 5 s^24–26^.

**Fig. 2:**
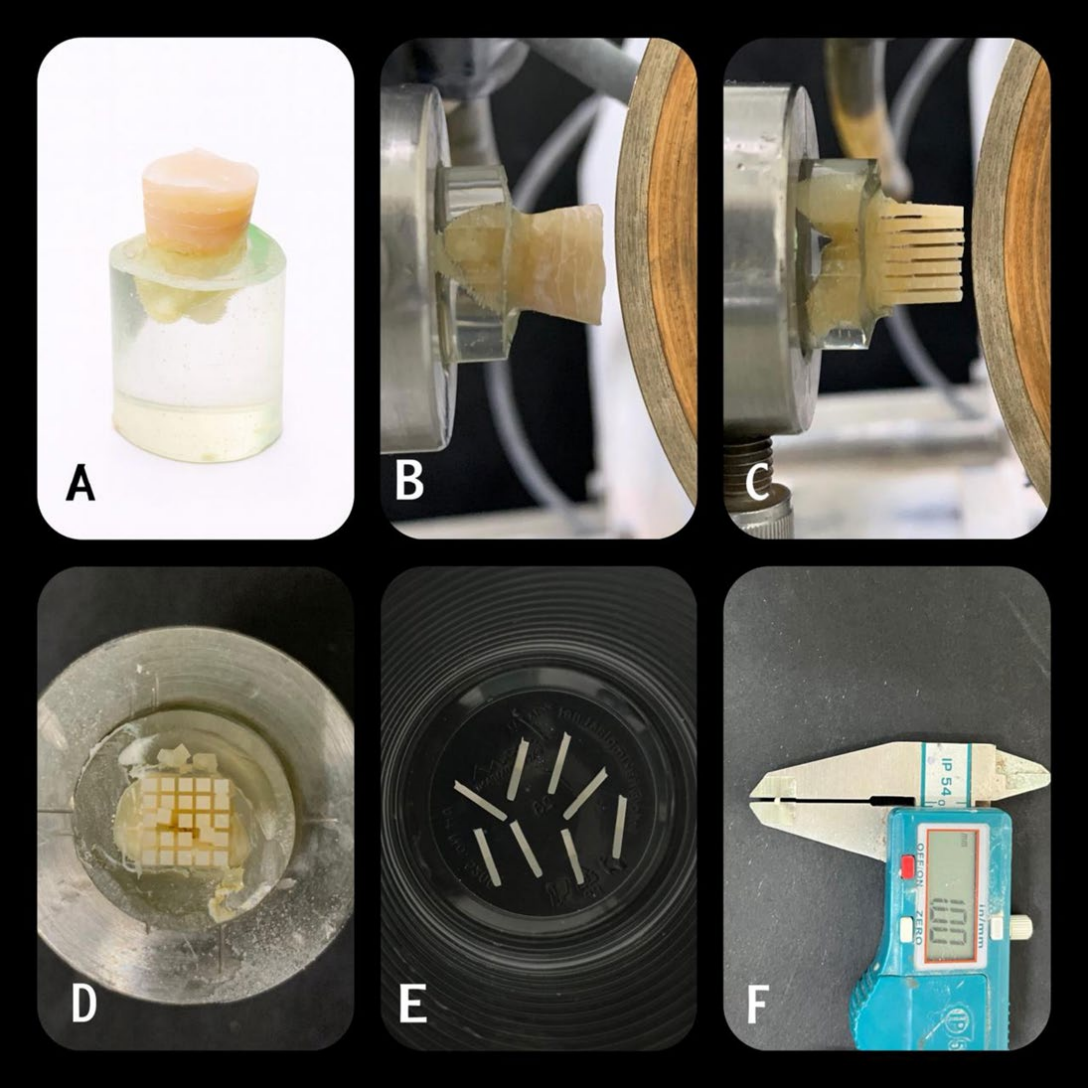
Steps of specimen’s preparation for microtensile bond strength, failure mode analysis and nanolekage tests

Total of eighty bonded teeth were sectioned in perpendicular and vertical directions along the “x” and “y” axes to produce rectangular beams measuring approximately 1 mm² in cross-sectional area and 5–7 mm in length, each containing composite, adhesive, and dentin (Fig. 2). Each tooth yielded approximately ten beams, and any beam with remaining enamel was excluded. A digital caliper (Model CD-6BS, Mitutoyo, Tokyo, Japan) was used to verify the cross-sectional measurements of each of the prepared beams^17^.

### Microtensile bond strength test (µTBS)

**Fig. 3:**
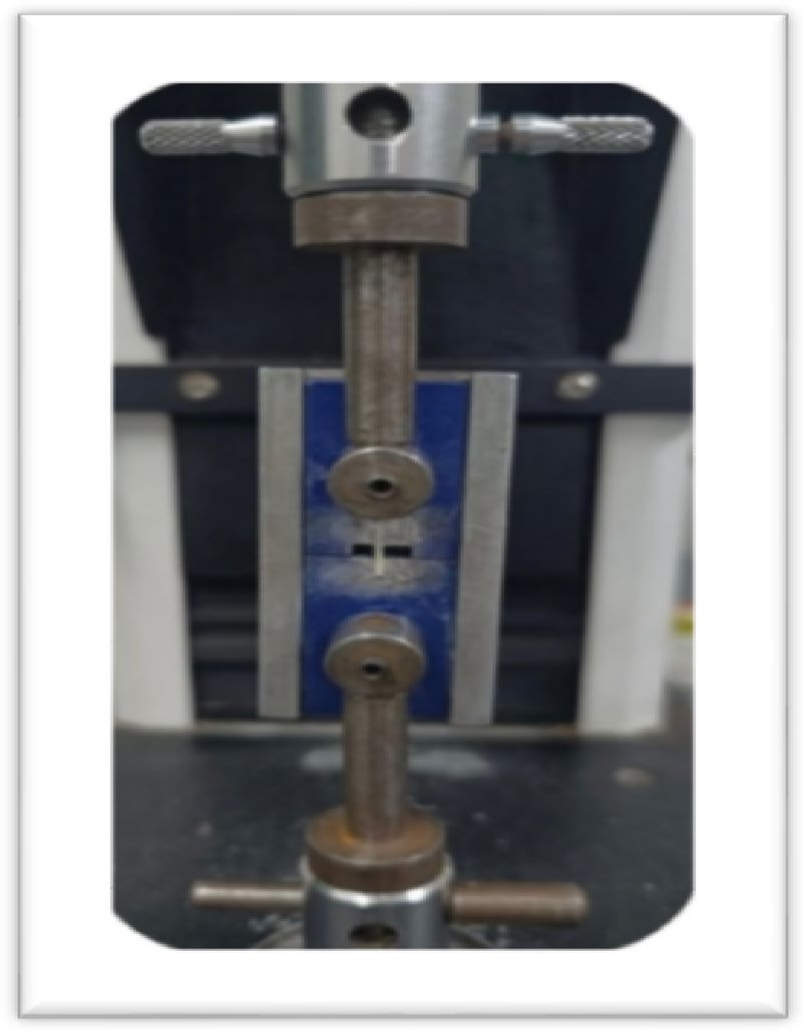
Fixation of the beam on the universal testing machine for micro-tensile bond strength testing.

Four beams from each tooth were fixed to a customized jig with cyanoacrylate glue (Zapitˏ Dental Ventures of America, Coronaˏ CAˏ USA) (Fig. 3). Tensile loading was applied using a universal testing machine (Instron, MA, USA) equipped with a 500 N load cell, and load was applied in at 1 mm/min crosshead speed till debonding occurred. The experimental and testing protocol used in the present study is consistent with the guidelines outlined in ISO/TS 4640:2023.ISO/TS 4640:2023^27^

## Failure mode analysis

A stereomicroscopic examination (Nikon MA 100, Tokyo, Japan) at 30× magnification was performed to inspect each fractured beam and identify the type of failure. Three categories were identified: (A) Cohesive: when fracture observed within the composite resin or within the dentin substrate. (B) Adhesive: when fracture observed at the bonding interface. (C) Mixed failure: when the fracture showed both adhesive and cohesive modes^28^.

## Nanoleakage evaluation

For nanoleakage assessment, three beams per tooth were randomly chosen. Both ends of each beam were painted with two coats of transparent varnish, leaving a 1-mm uncoated area from the adhesive interface. At first, the specimens were immersed in water for 10 min, then transferred to a 50% (w/v) ammoniacal silver nitrate solution and kept for 24 h in complete darkness. After a 5-minute rinse, the beams were placed in a photo-developing solution (Kodak GBX fixer and replenisher, Kodak, Rochester, NY, USA) and exposed to fluorescent light for 8 h to reduce the diamine silver ions within leakage channels to metallic silver grains^29^. Then, beams were rinsed and polished sequentially with 600–4000 grit silicon-carbide papers. Polishing was then performed using 6, 3, and 1 μm diamond suspensions (Diamat, Pace Technologies, Tucson, AZ, USA). The beams were cleansed ultrasonically and air-dried for 24 h. After that, imaging of the resin–dentin interface was performed using Field-Emission Scanning Electron Microscopy (FE-SEM) in backscattered mode at 20 kV, a 10-mm working distance, and 4000× magnification^17^.

### Micromorphological pattern analysis of the adhesive interface

**Fig. 4:**
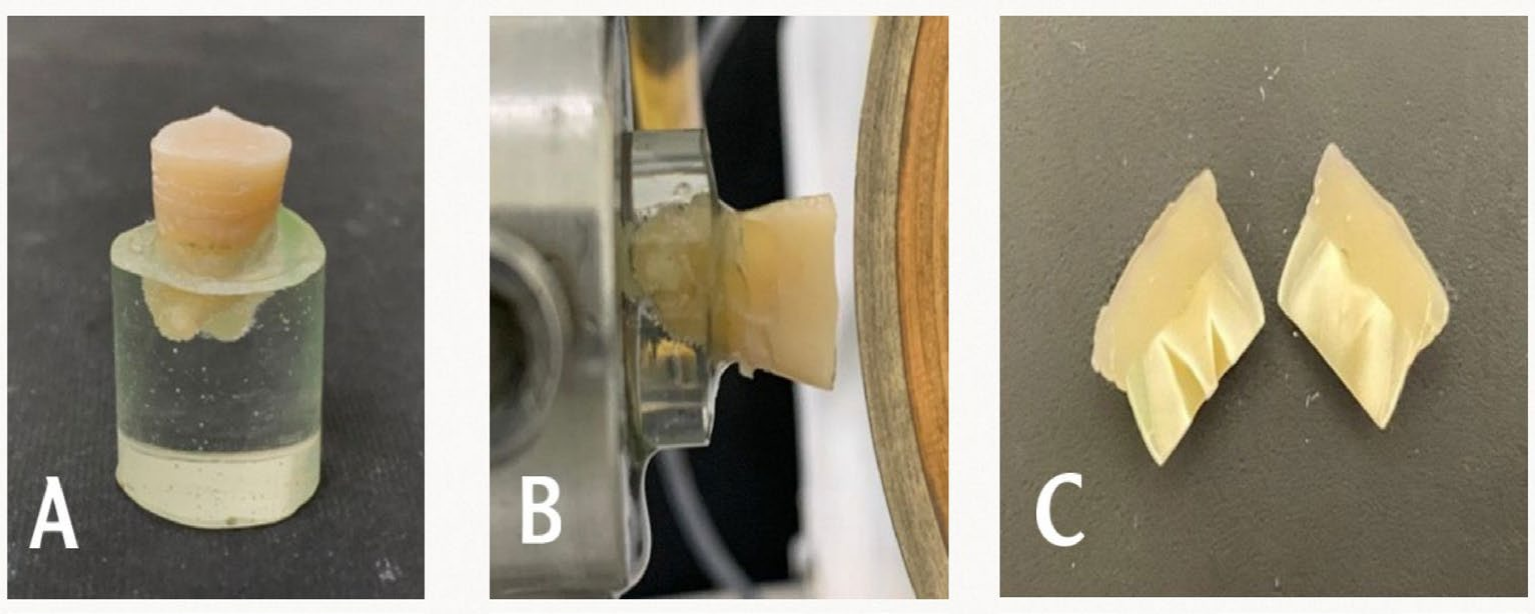
Steps of specimens sectioning for micromorphological surface analysis.

For the micromorphological evaluation, the remaining sixteen bonded teeth were cut mesiodistally with a diamond saw at low-speed under water cooling, producing two halves at a right angle to the resin–dentin interface (Fig. 4). The cut surfaces were subsequently polished under water using 600–4000 grit silicon-carbide papers, followed by final polishing with 6, 3, and 1 μm diamond pastes. After 10 min ultrasonic cleaning, the bonded area was treated with 10% orthophosphoric acid for 10 s. After that, immersed in 5% sodium hypochlorite for 5 min. The resin–dentin interface was then imaged using FE-SEM in secondary electron mode at 2000× magnification, 20 kV and a 10-mm working distance^30^.

### Statistical analysis

Data were analyzed using SPSS version 27.0 (SPSS Inc., Chicago, IL, USA). The data distribution was examined with Kolmogorov–Smirnov and Shapiro–Wilk tests, and values were presented as mean ± SD. Statistical significance was set at *p* < 0.05. The influence of the independent factors was assessed using a three-way ANOVA, followed by Tukey’s post hoc test for pairwise comparisons.

## Results

### Microtensile bond strength (µTBS)

The results of three-way ANOVA test demonstrated that surface treatment, adhesive mode, and aging protocol significantly influenced bond strength values and are presented in Table 1. The mean µTBS values for all groups are presented in Table 2. In the immediate condition, the highest µTBS value was recorded for the 0.3 wt% AL-treated ER group (37.88 ± 1.98 MPa), which was significantly higher than the other groups (*p* ≤ 0.05), followed by the 0.03 wt% AL-treated ER group and the control ER group. In the SE mode, the µTBS values were generally lower than those in the ER mode. However, the 0.3 wt% AL-treated group showed relatively higher bond strength (23.56 ± 1.46 MPa), with no statistically significant differences among the SE groups (*p* > 0.05).

Following thermocycling, µTBS values decreased across all groups. The 0.3 wt% AL ER group maintained the highest bond strength, followed by the 0.03 wt% AL and CHX ER groups. In the SE mode, the 0.3 wt% AL group continued to record relatively higher bond strength (23.74 ± 1.43 MPa) compared to other SE groups.

### Failure mode analysis

**Fig. 5:**
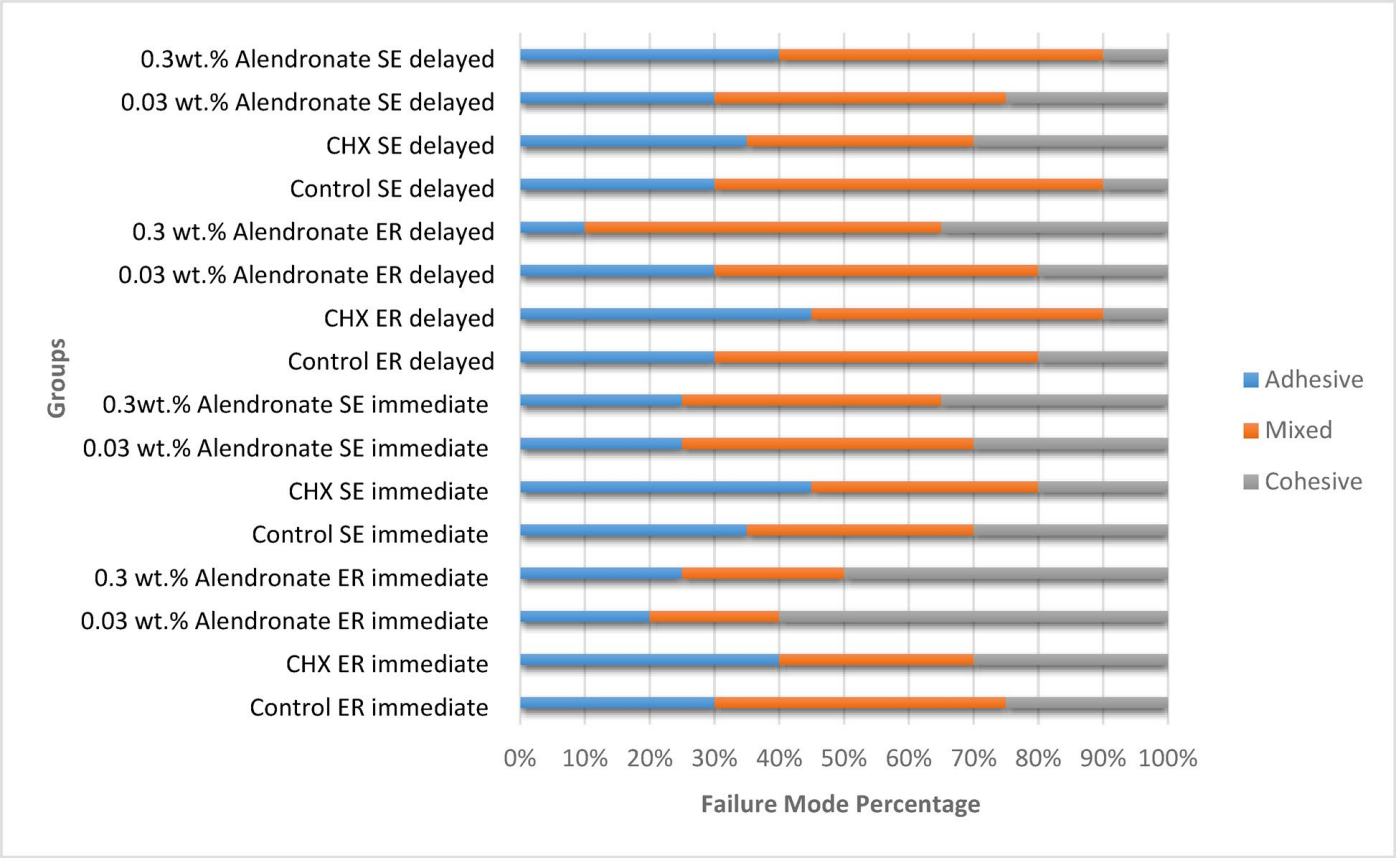
Mode of failure percentage of all tested groups.

**Fig. 6:**
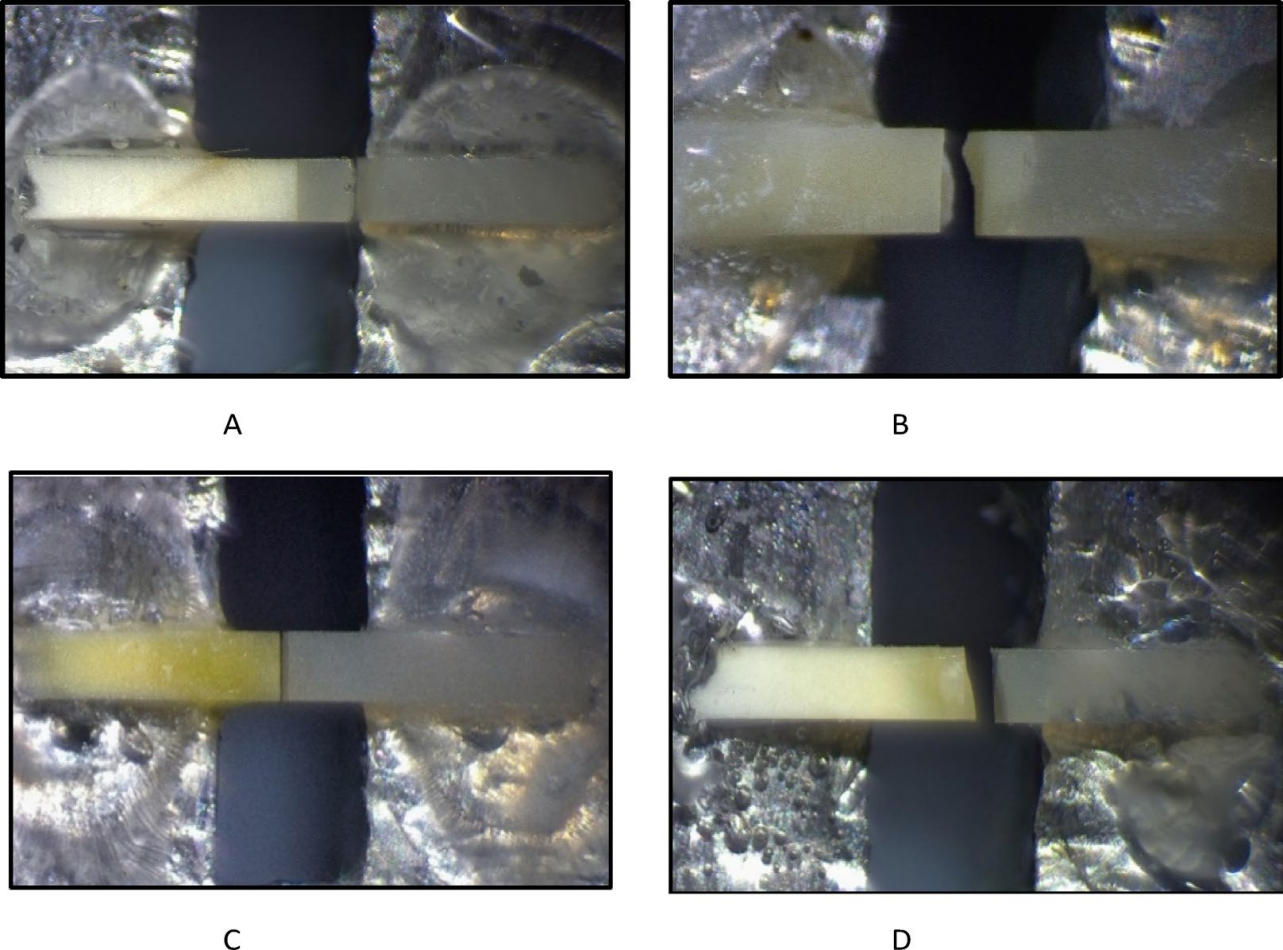
Stereomicroscope images showing (**A**): cohesive failure in composite, (**B**): cohesive failure in dentin, (**C**): adhesive failure, (**D**): mixed failure.

Failure mode of the fractured specimens are presented in Figs. 5 and 6. At the 24-hour time interval, stereomicroscope images revealed that the 0.3 wt% and 0.03 wt% AL-treated ER groups predominantly exhibited cohesive failures, whereas the control ER group showed a higher incidence of mixed failures, and the CHX-treated ER group predominantly demonstrated adhesive failures. In the SE groups, most failures were mixed. After thermocycling, AL-treated ER groups showed shift from cohesive to mixed failures. In SE groups, mixed failures remained the predominant mode even after aging.

**Table 1:** Three-way ANOVA results for microtensile bond strength comparisons among the groups.

Source	Type III sum of squares	Df	Mean square	F	Sig.
Corrected model	1534.913^a^	15	102.328	30.095	0.000
Intercept	51784.431	1	51784.431	15230.029	0.000
ST	272.706	3	90.902	26.735	0.000
Mode	814.060	1	814.060	239.419	0.000
Time	164.882	1	164.882	48.492	0.000
ST * mode	96.492	3	32.164	9.460	0.000
ST * time	60.179	3	20.060	5.900	0.001
Mode * time	77.336	1	77.336	22.745	0.000
ST * mode * time	49.259	3	16.420	4.829	0.004
Error	217.610	64	3.400		
Total	53536.954	80			
Corrected total	1752.523	79			

ST, Surface treatment; Mode, Adhesive mode; Time, Time of testing.

^a^R Squared = 0.876 (Adjusted R Squared = 0.847).

*P* value < 0.05.

**Table 2:** Mean microtensile bond strength (µTBS) values and standard deviations (SD) for the tested groups, expressed in megapascals (MPa).

After 24-hrs	Control	CHX	0.03wt.% AL	0.3 wt% AL
Etch- rinse	28.58 ± 1.61^b, c^	26.44 ± 1.15 ^c, d^	31.30 ± 1.87 ^b^	37.88 ± 1.98 ^a^
Self-etch	22.70 ± 2.15 ^d, e^	21.71 ± 0.99 ^e^	22.85 ± 1.20 ^d, e^	23.56 ± 1.46 ^d, e^
After thermocycling				
Etch-rinse	23.80 ± 2.13 ^d, e^	25.91 ± 1.92 ^c, d^	26.36 ± 2.23 ^c, d^	28.80 ± 2.65 ^b, c^
Self -etch	20.32 ± 1.76 ^e^	22.37 ± 1.42 ^d, e^	20.77 ± 2.59 ^e^	23.74 ± 1.43 ^d, e^

(*p*-values < 0.05)

Groups identified by different superscripts were significantly different at *p* < 0.05.

### Nanolekage evaluation

**Fig. 7:**
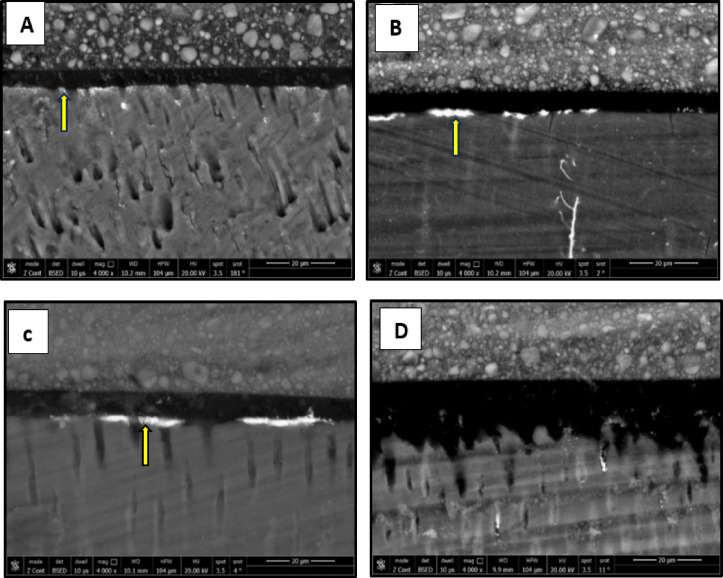
FE-SEM micrographs of immediate ER groups at 4000x magnification showing the nanolekage pattern of: (**A**): control group, (**B**): CHX group, (**C**): 0.03 wt% AL group and (**D**); 0.3 wt% AL group.

**Fig. 8:**
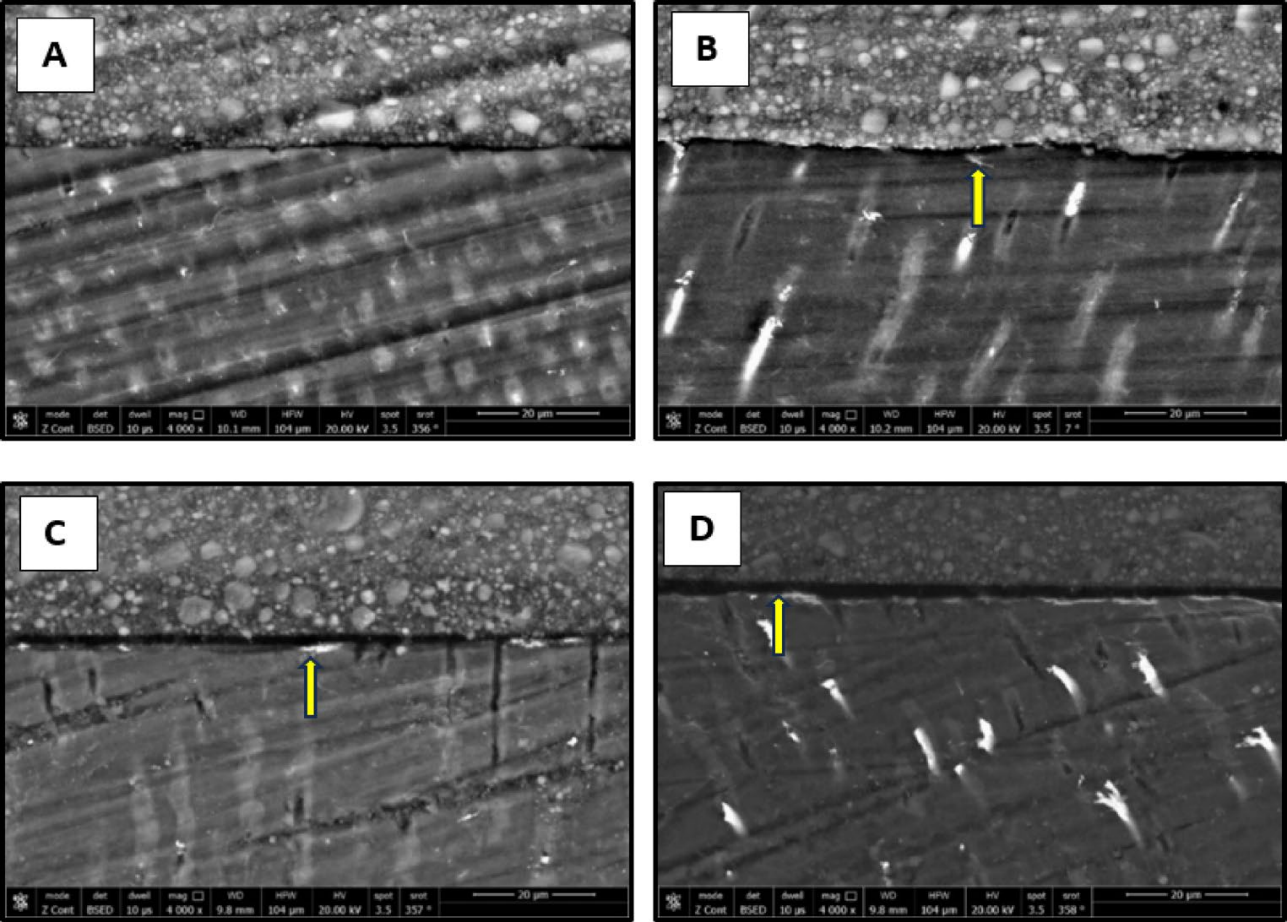
FE-SEM micrographs of immediate SE groups at 4000x magnification showing the nanolekage pattern of: (**A**): control group, (**B**): CHX group, (**C**): 0.03 wt% AL group and (**D**); 0.3 wt% AL group.

**Fig. 9:**
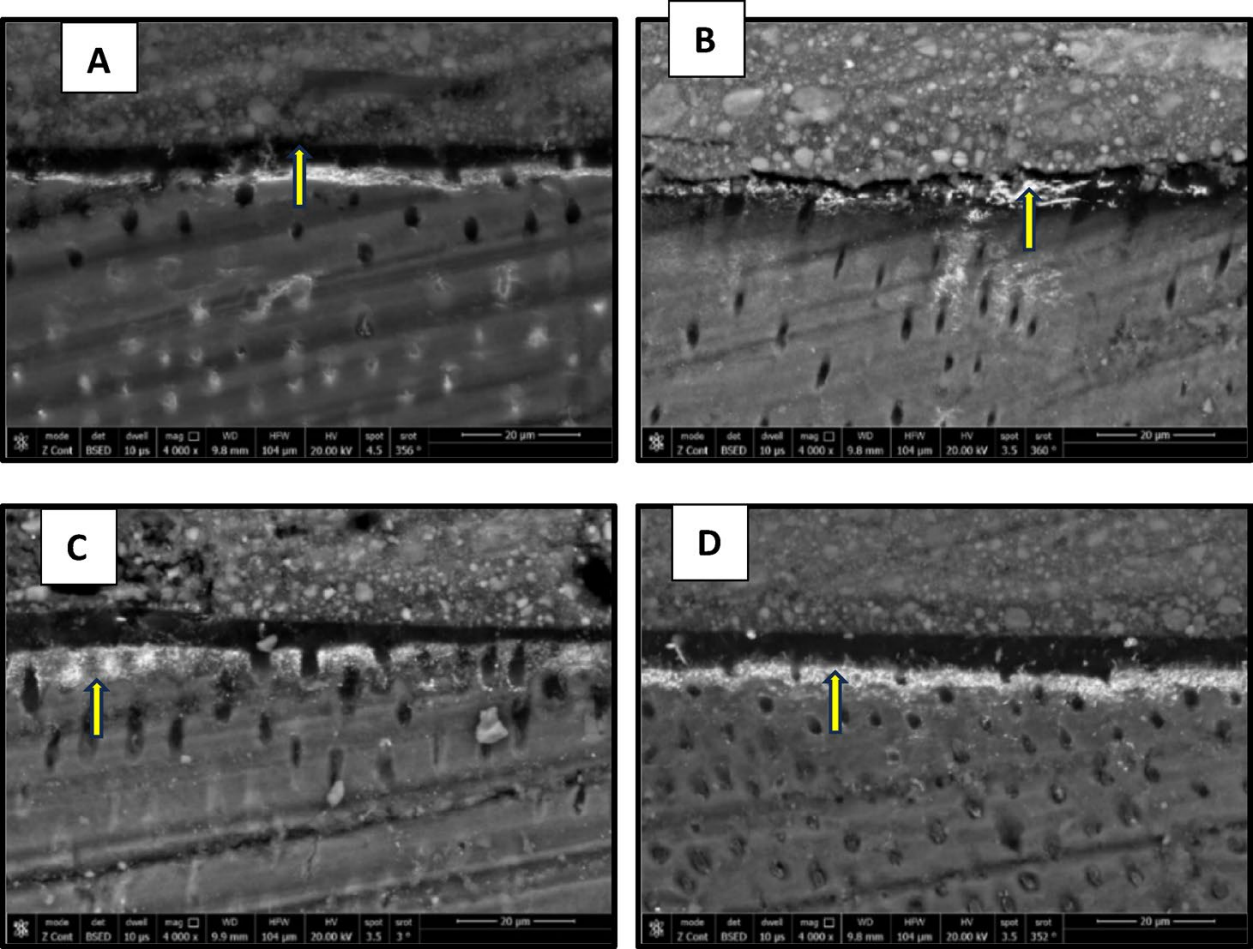
FE-SEM micrographs of aged ER groups at 4000x magnification showing the nanolekage pattern of: (**A**): control group, (**B**): CHX group, (**C**): 0.03 wt% AL group and (**D**):0.3 wt% group.

**Fig. 10:**
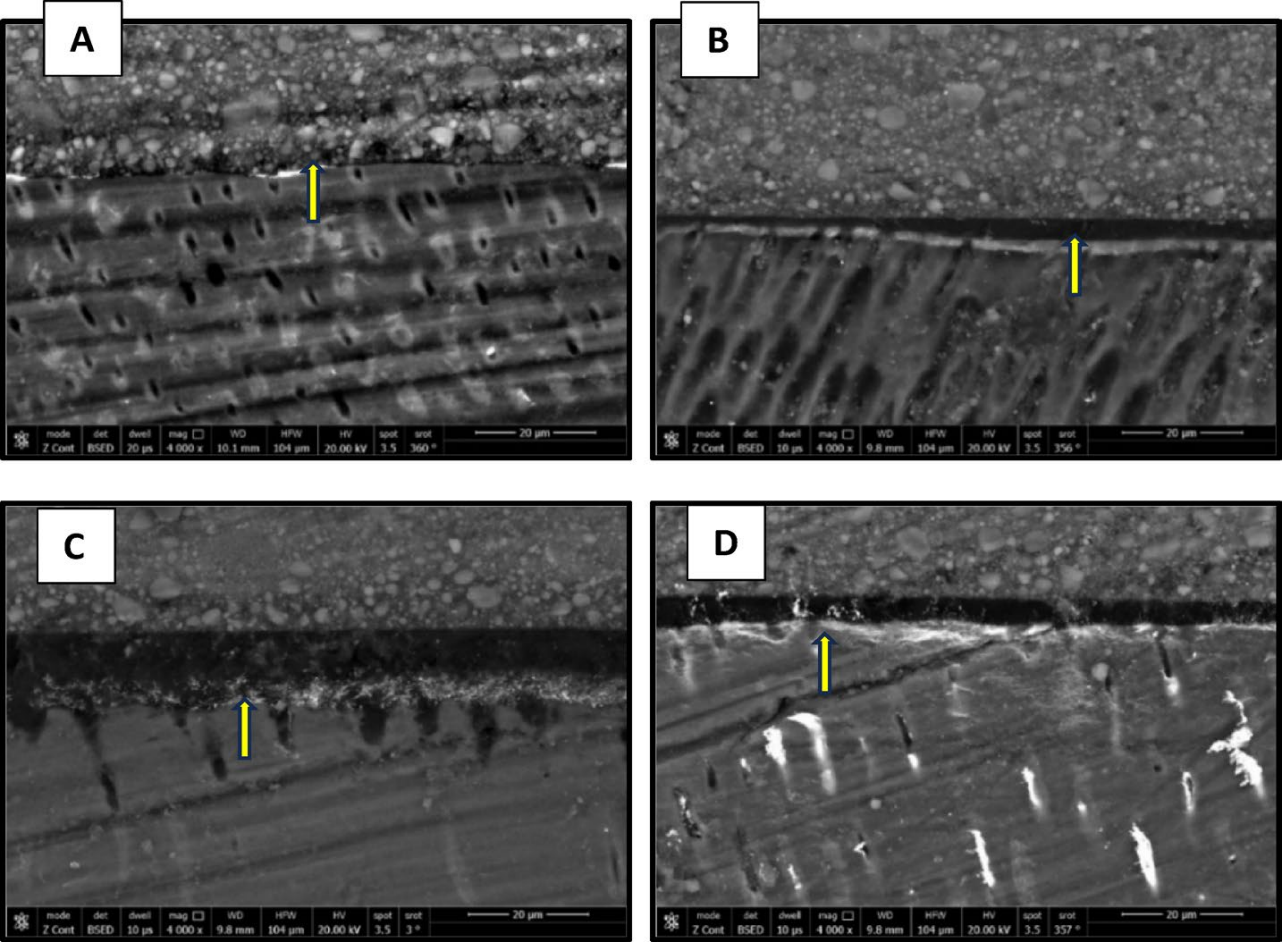
FE-SEM micrographs of aged SE groups at 4000x magnification showing the nanolekage pattern of: (**A**): control group, (**B**): CHX group, (**C**): 0.03 wt% AL group, and (**D**): 0.3 wt% AL group.

The results of nanoleake are presented in Figs. 7, 8, 9 and 10. FE-SEM imaging revealed that silver uptake was generally more pronounced in the etch-and-rinse (ER) groups than in the self-etch (SE) groups. FE-SEM micrographs of the immediate ER groups revealed the following: the control, the CHX and the 0.03 wt% AL groups showed a spotted pattern, while the 0.3 wt% AL group demonstrated nearly no silver uptake. In the immediate SE groups, the control group showed almost no silver uptake, while the CHX- and AL-treated groups exhibited a spotted pattern. In the aged ER groups, the control group and the 0.3 wt% AL group showed a water-tree pattern, while the CHX and 0.03 wt% AL groups displayed a reticular pattern. For the aged SE groups, the control group exhibited a slight spotted pattern, the CHX group displayed a reticular pattern, while both AL groups showed a water-tree pattern.

### Micromorphological analysis of the resin-dentin interface

**Fig. 11:**
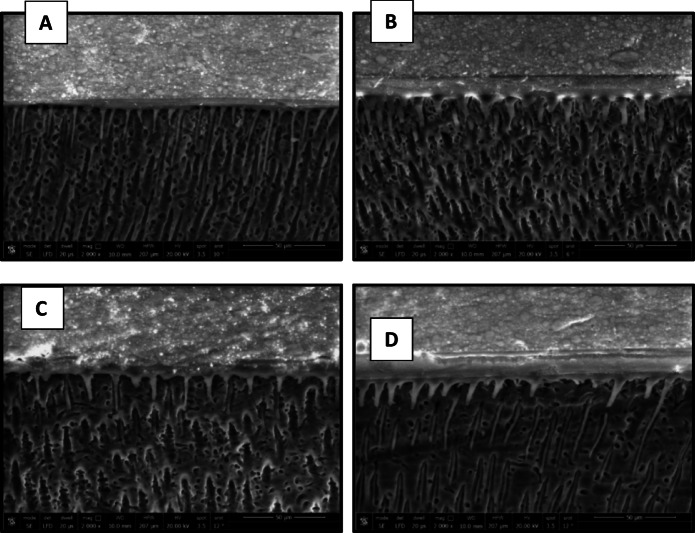
FE-SEM micrographs of immediate ER groups at 2000x magnification showing resin-dentin interface of: (**A**): control group, (**B**): CHX group, (**C**): 0.03 wt% AL group and (**D**):0.3wt%AL group.

**Fig. 12:**
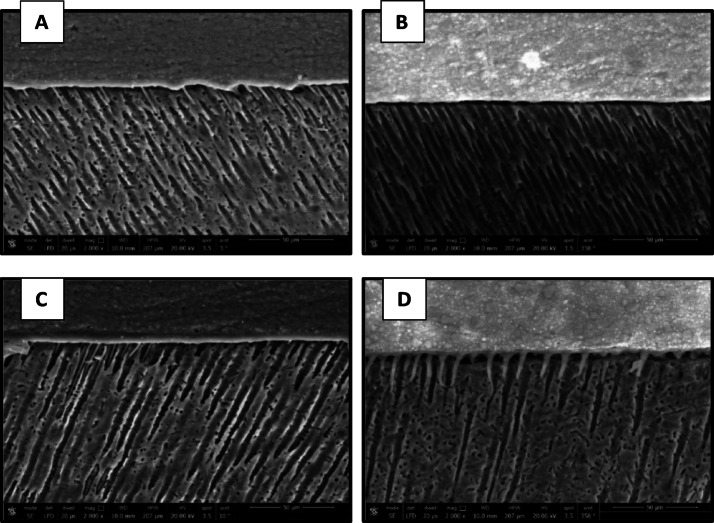
FE-SEM micrographs of the immediate SE groups at 2000x magnification showing resin-dentin interface of: (**A**): control group, (**B**): CHX group, (**C**): 0.03 wt% AL group and (**D**):0.3wt%AL group.

**Fig. 13:**
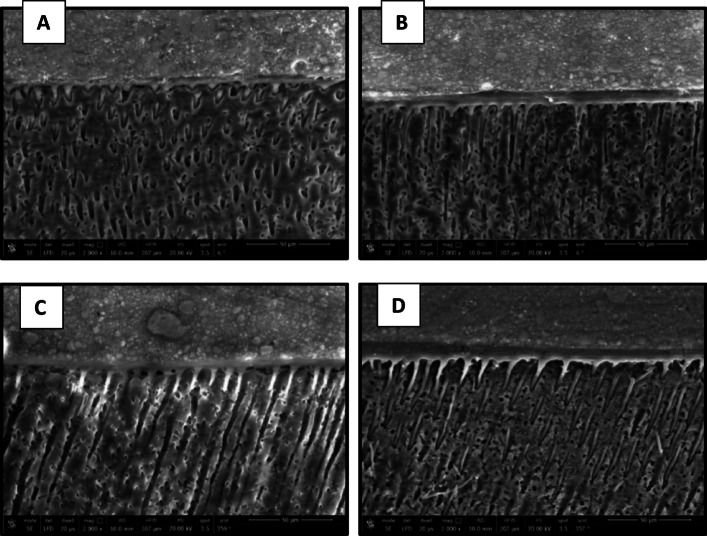
FE-SEM micrographs of aged ER groups at 2000x magnification showing resin-dentin interface of: (**A**): control group, (**B**): CHX group, (**C**): 0.03 wt% AL group and (**D**):0.3wt%AL group.

**Fig. 14:**
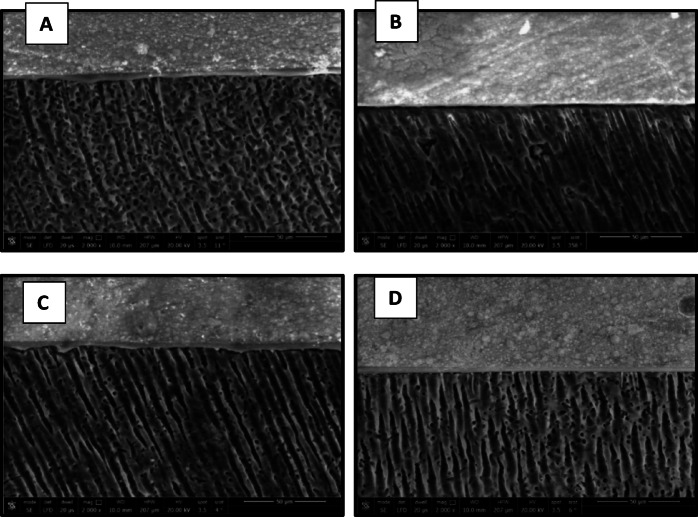
FE-SEM micrographs of aged SE groups at 2000x magnification showing resin-dentin interface of: (**A**): control group, (**B**): CHX group, (**C**): 0.03 wt% AL group showing and (**D**): 0.3 wt% AL group.

The results of micromorphological analysis of the resin-dentin interface are presented in Figs. 11, 12, 13 and 14. FE-SEM micrographs showed that in the immediate ER groups, the control group exhibited extensive resin tags with slight interfacial gaps, the CHX group showed short resin tags, the 0.03 wt% AL group displayed elongated and numerous resin tags, and the 0.3 wt% AL group displayed long, thick resin tags characterized by distinct funnel-shaped pattern extending deeply into the dentin, without signs of separation or interfacial gaps. In the immediate SE groups, the control, CHX and the 0.03 wt% AL groups showed thin and short resin tags while the 0.3 wt% AL group exhibited numerous resin tags. For the aged ER groups, the control and 0.3 wt% AL groups demonstrated numerous and extended resin tags, whereas the CHX group and 0.03 wt% AL group showed short, non-extended resin tags. In the aged SE groups, all groups exhibited thin and short resin tags penetrating the dentin surface, with minor interfacial gaps and a relatively thin hybrid layer.

## Discussion

Achieving durable resin–dentin bonding is still a major challenge in restorative dentistry, mainly due to hybrid layer breakdown triggered by endogenous matrix metalloproteinases (MMPs). Chlorhexidine (CHX) is the most widely investigated MMP inhibitor used to enhance resin–dentin bond stability^17^. However, its effectiveness is limited by gradual leaching from the hybrid layer, which reduces its long-term protective effects^15^. Emerging alternatives that offer greater stability and sustained MMP-inhibiting activity are of considerable interest. Sodium alendronate (AL) could be one of these alternatives, as it shows promising properties. It can inhibit MMPs, bind strongly to calcium, and stabilize the collagen matrix^31,32^. However, there is limited evidence supporting its application in adhesive dentistry, particularly regarding its interaction with universal adhesives used in ER or SE modes. Thus, the current study aimed to evaluate the impact of dentin pre-treatment with sodium alendronate solution (0.03 wt% and 0.3 wt%) on the bonding performance of a universal adhesive.

Based on the findings of the study, the null hypothesis stating that pre-bond treatment with alendronate does not affect the bond strength, regardless of etching mode or aging period, was rejected.

The findings of the current study demonstrated that the etch-and-rinse groups had significantly greater mean bond strength values than the self-etch groups. This may be related to the ability of phosphoric acid etchant to completely remove the smear layer, creating a demineralized dentin zone of approximately 5–8 μm. This could permit better resin monomer penetration, promoting micromechanical interlocking within the dentinal tubules and the formation of longer, well-defined resin tags, thereby enhancing bond strength^33–35^. Conversely, the relatively mild acidity of self-etch adhesives results in minimal demineralization and the formation of thin hybrid layers with shallow resin infiltration, which may explain their lower µTBS values^36^.

Regarding surface treatment, alendronate (AL)-treated groups demonstrated significantly higher µTBS values in comparison to the other groups. This suggests a potential modifying effect of alendronate on dentin collagen. Besides alendronate’s ability to inhibit MMPs, it can also form ester linkages between its hydroxyl groups and the carboxyl groups of collagen. This could stabilize the collagen matrix, which in turn improves the bond between resin and dentin.^37–39^ In the current study, the mean bond strength of 0.3 wt% AL-treated groups was significantly higher than those of 0.03 wt% AL groups, suggesting that the effect is concentration-dependent, with 0.3 wt% being more effective in enhancing the hybrid layer. This enhancement may be related to the inhibitory influence of alendronate on matrix metalloproteinases (MMPs). Sun et al ^29^ reported that 0.03 wt% alendronate only partially suppresses MMP-2 activity, whereas 0.3 wt% completely inhibits it. Many studies^39–41^ on human bone support these results, confirming that the inhibitory effect of alendronate is concentration-dependent.

After 24 h, CHX- treated groups showed relatively lower bond strength. This finding aligns with previous studies^42–45^, which also indicated diminished initial bond strength when CHX was applied with MDP-containing universal adhesives. This reduction may be due to CHX cations binding to phosphate and calcium in hydroxyapatite, thereby disrupting MDP–calcium salt formation and interfering with adhesive performance^46–48^.

Following thermocycling, all groups exhibited reduced bond strength relative to their 24-hour values. This reduction was particularly pronounced in the ER groups, where it may be attributed to biodegradation of the hybrid layer. Acid etching widens the dentinal tubule orifices, removes minerals, and exposes a collagen network. Exposed collagen that is not fully infiltrated with resin becomes more liable to enzymatic and hydrolytic degradation. Additionally, the polymer’s ability to absorb water speeds up the hydrolysis of the resin matrix, which weakens the adhesive interface^49,50^.

Regarding mode of failure, after thermocycling, Alendronate ER groups showed a shift from cohesive failure to mixed failure. This shift indicates a reduction in bond strength. In the SE groups, mixed failures remained the predominant failure mode even after aging. The findings align with the µTBS results and with previous studies^27,51^, which demonstrated that cohesive failures are associated with higher bond strength, while adhesive and mixed failures correspond to lower bond strength.

Nanoleakage evaluation of the current study confirmed that silver deposition was present along the resin–dentin interface approximately in all groups, indicating that interfacial defects existed irrespective of the bonding strategy. Nanoleakage was more evident in the ER groups compared to the SE groups. This finding is in agreement with previous studies^52–54^, which similarly observed greater nanoleakage with etch-and-rinse strategy. This increased nanoleakage is likely due to extensive demineralization and greater collagen matrix exposure, which is often incompletely infiltrated by adhesive resin, leaving channels for fluid movement and silver ion penetration. Notably, the immediate 0.3 wt% alendronate ER group displayed minimal silver uptake, suggesting that alendronate pre-bond treatment may enhance initial sealing of the adhesive interface by chemically interacting with calcium in hydroxyapatite and stabilizing collagen fibrils^55^.

Micromorphological analysis of the current study aligns with a previous study^56^, that demonstrated a positive correlation between the bond strength and the length of resin tags. All SE groups showed thinner hybrid layers with small interfacial gaps, which could explain their comparatively lower bond strengths. In the immediate ER groups, control specimens showed well-formed resin tags with minor interfacial gaps, whereas the CHX group exhibited shorter resin tags, consistent with its lower µTBS values. Both AL-treated groups demonstrated enhanced resin infiltration, especially 0.3 wt%, which displayed long, thick, funnel-shaped resin tags and a more prominent hybrid layer. The improved micromorphology in the AL groups could be attributed to its chemical structure, which includes two phosphonate groups that strongly attract calcium ions found in the tooth substrate^57,58^. Furthermore, MDP molecules in the universal adhesive may form hydrogen bonds between their phosphate groups and hydroxyl groups of alendronate. This interaction could act as a chemical bridge, facilitating the formation of a stable calcium–alendronate–MDP complex and thereby strengthening the adhesive interface between MDP and sodium alendronate. Additionally, the hydrophilic nature of sodium alendronate may enhance its compatibility with the hydrophilic monomers of the adhesive system, facilitating deeper infiltration and more durable bonding^58,59^.

These findings suggest that dentin pre-bond treatment with alendronate solution, particularly the 0.3 wt% concentration, represents a promising strategy for enhancing bond strength. Although yielding promising outcomes, the study has several limitations. Being an in vitro, it cannot fully simulate the oral environment which includes mechanical loading, bacterial interactions, pH fluctuations, and enzymatic activity. The artificial aging protocol utilized was limited to 5,000 thermocycles, which may not sufficiently replicate long-term clinical aging. Qualitative nanoleakage evaluation relies on subjective visual assessment, which could lead to bias and reduced reproducibility. For a more accurate evaluation, this method should be complemented with quantitative analysis. Further investigations are required to integrate both qualitative and quantitative nanoleakage analyses, employ extended aging protocols, incorporate mechanical fatigue loading, MMPs activity assay as well as comprehensive biocompatibility assessments. Such investigations would provide a more complete understanding of the sealing efficacy and durability of sodium alendronate and allow a more accurate evaluation of its long-term clinical applicability as a dentin pre-bond treatment strategy.

## Conclusions

Dentin treatment with alendronate specifically 0.3 wt%, before bonding, appeared to enhance bond strength, particularly with etch-and-rinse mode. The results of failure mode analysis, nanoleakage evaluation and micromorphological interface observations confirmed these findings.

## Data Availability

The datasets used and/or analyzed during the current study are available from the corresponding author on reasonable request.

## Abbreviations

10-MDP: 10-methacryloyloxydecyl dihydrogen phosphate
AL: Alendronate
ANOVA: Analysis of variance
CHX: Chlorhexidine digluconate
ER: Etch-and-rinse
SE: Self-etch
MMPs: Matrix metalloproteinases
µTBS: Micro-tensile bond strength test
SEM: Scanning electron microscopy
FE-SEM: Field emission scanning electron microscopy

## References

[1] Jang, J. H. et al. Comparative study of the dentin bond strength of a new universal adhesive. *Dent. Mater. J.* **35**, 606–612. 10.4012/dmj.2015-422 (2016). 27477226 10.4012/dmj.2015-422

[2] Manfroi, F. B. et al. Bond strength of a novel one bottle multi-mode adhesive to human dentin after six months of storage. *open. dentistry J.* **10**, 268–277. 10.2174/1874210601610010268 (2016). 10.2174/1874210601610010268 PMC4901199 27347230

[3] Tjäderhane, L. et al. Optimizing dentin bond durability: Control of collagen degradation by matrix metalloproteinases and cysteine cathepsins. *Dent. Mater.* **29**, 116–135. 10.1016/j.dental.2012.08.004 (2013). 22901826 10.1016/j.dental.2012.08.004 PMC3684081

[4] Frassetto, A. et al. Mechanisms of degradation of the hybrid layer in adhesive dentistry and therapeutic agents to improve bond durability–a literature review. *Dent. materials: official publication Acad. Dent. Mater.* **32**, e41–53. 10.1016/j.dental.2015.11.007 (2016). 10.1016/j.dental.2015.11.007 26743967

[5] Zhang, S. C. & Kern, M. The role of host-derived dentinal matrix metalloproteinases in reducing dentin bonding of resin adhesives. *Int. J. Oral Sci.* **1**, 163–176. 10.4248/ijos.09044 (2009). 20690420 10.4248/IJOS.09044 PMC3470104

[6] Sulkala, M. et al. Matrix metalloproteinase-8 (MMP-8) is the major collagenase in human dentin. *Arch. Oral Biol.* **52**, 121–127. 10.1016/j.archoralbio.2006.08.009 (2007). 17045563 10.1016/j.archoralbio.2006.08.009

[7] Breschi, L. et al. Use of a specific MMP-inhibitor (galardin) for preservation of hybrid layer. *Dent. Mater.* **26**, 571–578 (2010). 20299089 10.1016/j.dental.2010.02.007 PMC3881003

[8] da Silva, E. M., de Sá Rodrigues, C. U., de Oliveira Matos, M. P., de Carvalho, T. R. & dos Santos, G. B. Experimental etch-and-rinse adhesive systems containing MMP-inhibitors: Physicochemical characterization and resin-dentin bonding stability. *J. Dent.* **43**, 1491–1497. 10.1016/j.jdent.2015.10.004 (2015). 26456899 10.1016/j.jdent.2015.10.004

[9] Anumula, L., Ramesh, S. & Kolaparthi, V. S. K. Matrix metalloproteinases in dentin: Assessing their presence, activity, and inhibitors - a review of current trends. *Dent. Mater.* **40**, 2051–2073. 10.1016/j.dental.2024.09.011 (2024). 39368893 10.1016/j.dental.2024.09.011

[10] Breschi, L. et al. The evolution of adhesive dentistry: From etch-and-rinse to universal bonding systems. *Dent. Mater.* **41**, 141–158. 10.1016/j.dental.2024.11.011 (2025). 39632207 10.1016/j.dental.2024.11.011

[11] Poppolo Deus, F. & Ouanounou, A. Chlorhexidine in dentistry: Pharmacology, uses, and adverse effects. *Int. Dent. J.* **72**, 269–277. 10.1016/j.identj.2022.01.005 (2022). 35287956 10.1016/j.identj.2022.01.005 PMC9275362

[12] Giacomini, M. C. et al. Profile of a 10-MDP-based universal adhesive system associated with chlorhexidine. *J. Mech. Behav. Biomed. Mater.*10.1016/j.jmbbm.2020.103925 (2020). 10.1016/j.jmbbm.2020.103925 32957220

[13] Kast, R. E. Potential benefits of adding alendronate, celecoxib, itraconazole, ramelteon, and simvastatin to endometrial cancer treatment: The EC5 Regimen. *Curr. Issues. Mol. Biol.*10.3390/cimb47030153 (2025). 10.3390/cimb47030153 PMC11941490 40136407

[14] Heikkilä, P. et al. Bisphosphonates inhibit stromelysin-1 (MMP-3), matrix metalloelastase (MMP-12), collagenase-3 (MMP-13) and enamelysin (MMP-20), but not urokinase-type plasminogen activator, and diminish invasion and migration of human malignant and endothelial cell lines. *Anti-cancer drugs*. **13**, 245–254. 10.1097/00001813-200203000-00006 (2002). 11984068 10.1097/00001813-200203000-00006

[15] de Moraes, I. Q. S. et al. Inhibition of matrix metalloproteinases: a troubleshooting for dentin adhesion. *Restor. dentistry endodontics*. **45**, e31. 10.5395/rde.2020.45.e31 (2020). 10.5395/rde.2020.45.e31 PMC7431940 32839712

[16] Popov, K. et al. Synthesis, structures, properties, medical and industrial applications. *J. Mol. Liq.* **351**, 118619. 10.1016/j.molliq.2022.118619 (2022).

[17] Bedir, M. G. A., Karadas, M. & Bedir, F. Effect of matrix metalloproteinase inhibitors on bonding durability of universal adhesives. *Dent. Mater. J.* **42**, 581–590. 10.4012/dmj.2022-282 (2023). 37302822 10.4012/dmj.2022-282

[18] Fernandes, A. B. F. et al. Influence of two carbodiimides on the bond strength of universal adhesives to dentin. *Odontology* **110**, 99–105. 10.1007/s10266-021-00642-z (2022). 34279762 10.1007/s10266-021-00642-z

[19] Vachal, P. et al. Synthesis and study of alendronate derivatives as potential prodrugs of alendronate sodium for the treatment of low bone density and osteoporosis. *J. Med. Chem.* **49**, 3060–3063. 10.1021/jm060398v (2006). 16722624 10.1021/jm060398v

[20] Salim Al-Ani, A. A., Mutluay, M., Stape, T. H. S., Tjäderhane, L. & Tezvergil-Mutluay, A. Effect of various dimethyl sulfoxide concentrations on the durability of dentin bonding and hybrid layer quality. *Dent. Mater. J.* **37**, 501–505. 10.4012/dmj.2017-213 (2018). 29593164 10.4012/dmj.2017-213

[21] Alhenaki, A. M. et al. Dentin bond integrity of filled and unfilled resin adhesive enhanced with silica nanoparticles-an SEM, EDX, Micro-Raman, FTIR and micro-tensile bond strength study. *Polymers* **13**10.3390/polym13071093 (2021). 10.3390/polym13071093 PMC8037508 33808159

[22] Sebold, M. et al. Bonding interface and dentin enzymatic activity of two universal adhesives applied following different etching approaches. *Dent. Mater.* **38**, 907–923. 10.1016/j.dental.2022.03.001 (2022). 35289283 10.1016/j.dental.2022.03.001

[23] Hamouda, I. M., Samra, N. R. & Badawi, M. F. Microtensile bond strength of etch and rinse versus self-etch adhesive systems. *J. Mech. Behav. Biomed. Mater.* **4**, 461–466. 10.1016/j.jmbbm.2010.12.007 (2011). 21316634 10.1016/j.jmbbm.2010.12.007

[24] Eliasson, S. T. & Dahl, J. E. Effect of thermal cycling on temperature changes and bond strength in different test specimens. *Biomaterial investigations dentistry*. **7**, 16–24. 10.1080/26415275.2019.1709470 (2020). 10.1080/26415275.2019.1709470 PMC7033714 32128509

[25] Gale, M. S. & Darvell, B. W. Thermal cycling procedures for laboratory testing of dental restorations. *J. Dent.* **27**, 89–99. 10.1016/s0300-5712(98)00037-2 (1999). 10071465 10.1016/s0300-5712(98)00037-2

[26] bin Husein, A., Seoudi, S. M. F., El-Damanhoury, H. M. & Aziz, I. M. Abou Neel, EA. Hybrid layer, shear bond strength, and fracture patterns of titanium dioxide–doped phosphate glass–filled universal dental adhesives. *Eur. J. Gen. Dentistry*. **14**, 044–050 (2025).

[27] Santander-Rengifo, F. et al. Microtensile bond strength and failure mode of different universal adhesives on human dentin. *Int. Dent. J.* **74**, 1239–1247. 10.1016/j.identj.2024.04.009 (2024). 38734514 10.1016/j.identj.2024.04.009 PMC11551578

[28] Alrafee, S. A., Abdelgawad, A., Ali, M. A. S. & Tamimi, S. Bond strength of resin cement following biomimetic remineralization: An in vitro study. *Open. Dentistry J.* **18**. 10.2174/0118742106284569240227095212 (2024).

[29] Basha, I. Assessment of nano-leakage for three adhesive systems: An in vitro study. *Al-Azhar Assiut Dent. J.* **4**, 109–116. 10.21608/aadj.2021.206571 (2021).

[30] Hamama, H., Yiu, C. & Burrow, M. F. Effect of chemomechanical caries removal on bonding of resin-modified glass ionomer cement adhesives to caries-affected dentine. *Aust. Dent. J.* **60**, 190–199. 10.1111/adj.12318 (2015). 25989193 10.1111/adj.12318

[31] Henneman, Z. J., Nancollas, G. H., Ebetino, F. H., Russell, R. G. & Phipps, R. J. Bisphosphonate binding affinity as assessed by inhibition of carbonated apatite dissolution in vitro. *J. Biomed. Mater. Res. A*. **85**, 993–1000. 10.1002/jbm.a.31599 (2008). 17907244 10.1002/jbm.a.31599 PMC2743543

[32] Sun, J., Song, F., Zhang, W., Sexton, B. E. & Windsor, L. J. Effects of alendronate on human osteoblast-like MG63 cells and matrix metalloproteinases. *Arch. Oral Biol.* **57**, 728–736. 10.1016/j.archoralbio.2011.12.007 (2012). 22251575 10.1016/j.archoralbio.2011.12.007

[33] Zheng, P., Zaruba, M., Attin, T. & Wiegand, A. Effect of different matrix metalloproteinase inhibitors on microtensile bond strength of an etch-and-rinse and a self-etching adhesive to dentin. *Oper. Dent.* **40**, 80–86. 10.2341/13-162-l (2015). 24815915 10.2341/13-162-L

[34] Monteiro, T. M. A., Basting, R. T., Turssi, C. P., França, F. M. G. & Amaral, F. L. B. Influence of natural and synthetic metalloproteinase inhibitors on bonding durability of an etch-and-rinse adhesive to dentin. *Int. J. Adhes. Adhes.* **47**, 83–88. 10.1016/j.ijadhadh.2013.09.020 (2013).

[35] Pashley, D. H. et al. State of the art etch-and-rinse adhesives. *Dent. Mater.* **27**, 1–16 (2011). 21112620 10.1016/j.dental.2010.10.016 PMC3857593

[36] Lohbauer, U., Nikolaenko, S. A., Petschelt, A. & Frankenberger, R. Resin tags do not contribute to dentin adhesion in self-etching adhesives. *J. Adhes. Dent.* **10**, 97–103 (2008). 18512506

[37] Zhang, L. et al. Alendronate and polyelectrolyte synergically induce biomimetic mineralization of collagen and demineralized dentin. *Int. J. Biol. Macromol.* **308**, 142402. 10.1016/j.ijbiomac.2025.142402 (2025). 40154709 10.1016/j.ijbiomac.2025.142402

[38] Drake, M. T., Clarke, B. L. & Khosla, S. Bisphosphonates: mechanism of action and role in clinical practice. *Mayo Clinic proceedings* 83, 1032–1045. (2008). 10.4065/83.9.1032 10.4065/83.9.1032 PMC2667901 18775204

[39] Lai, T. J. et al. Alendronate inhibits cell invasion and MMP-2 secretion in human chondrosarcoma cell line. *Acta Pharmacol. Sin.* **28**, 1231–1235. 10.1111/j.1745-7254.2007.00607.x (2007). 17640487 10.1111/j.1745-7254.2007.00607.x

[40] Krüger, T. B., Syversen, U., Herlofson, B. B., Lian, A. M. & Reseland, J. E. Targeting a therapeutically relevant concentration of alendronate for in vitro studies on osteoblasts. *Acta Odontol. Scand.* **80**, 619–625. 10.1080/00016357.2022.2072522 (2022). 35605138 10.1080/00016357.2022.2072522

[41] Hedvičáková, V., Žižková, R., Buzgo, M., Rampichová, M. & Filová, E. The effect of alendronate on osteoclastogenesis in different combinations of M-CSF and RANKL growth factors. *Biomolecules* **11**10.3390/biom11030438 (2021). 10.3390/biom11030438 PMC8035832 33809737

[42] Ebrahimi-Chaharom, M. E., Moeinian, A., Abed-Kahnamouei, M., Daneshpooy, M. & Bahari, M. Effect of matrix metalloproteinase inhibitors on the dentin bond strength and durability of a two-step universal adhesive system. *J. Clin. experimental dentistry*. **17**, e233–e238. 10.4317/jced.62406 (2025). 10.4317/jced.62406 PMC11994208 40231141

[43] Lenzi, T. L., Tedesco, T. K., Soares, F. Z. & Loguercio, A. D. Rocha, R. O. Chlorhexidine does not increase immediate bond strength of etch-and-rinse adhesive to caries-affected dentin of primary and permanent teeth. *Braz. Dent. J.* **23**, 438–442. 10.1590/s0103-64402012000400022 (2012). 23207863 10.1590/s0103-64402012000400022

[44] García, A., López Jordi, M. C., Fabruccini, A. & Liberman, J. J. O. Effect of chlorhexidine pretreatment on demineralized dentin bond strength. *Odontoestomatología* 25 (2023).

[45] Ebrahimi, M., Majidinia, S. & Sarraf, A. Effect of chlorhexidine on immediate and delayed bond strength between resin and dentin of primary teeth: A systematic review and meta-analysis. *Front. dentistry*. **19**, 39. 10.18502/fid.v19i39.11749 (2022). 10.18502/fid.v19i39.11749 PMC9976634 36873616

[46] Morresi, A. L. et al. Thermal cycling for restorative materials: does a standardized protocol exist in laboratory testing? A literature review. *J. Mech. Behav. Biomed. Mater.* **29**, 295–308. 10.1016/j.jmbbm.2013.09.013 (2014). 24135128 10.1016/j.jmbbm.2013.09.013

[47] Shen, J. et al. Evaluation of the interaction of chlorhexidine and MDP and its effects on the durability of dentin bonding. *Dent. Mater.* **36**, 1624–1634. 10.1016/j.dental.2020.10.006 (2020). 33183774 10.1016/j.dental.2020.10.006

[48] Shafiei, F., Alikhani, A. & Alavi, A. A. Effect of chlorhexidine on bonding durability of two self-etching adhesives with and without antibacterial agent to dentin. *Dent. Res. J.* **10**, 795–801 (2013). PMC3872633 24379870

[49] Hashimoto, M., Fujita, S., Nagano, F., Ohno, H. & Endo, K. Ten-years degradation of resin-dentin bonds. *Eur. J. Oral. Sci.* **118**, 404–410. 10.1111/j.1600-0722.2010.00744.x (2010). 20662915 10.1111/j.1600-0722.2010.00744.x

[50] Pfeifer, C. S., Lucena, F. S. & Tsuzuki, F. M. Preservation strategies for interfacial integrity in restorative dentistry: A non-comprehensive literature review. *J. Funct. biomaterials*. **16**10.3390/jfb16020042 (2025). 10.3390/jfb16020042 PMC11856648 39997576

[51] Can-Karabulut, D. C., Oz, F. T., Karabulut, B., Batmaz, I. & Ilk, O. Adhesion to primary and permanent dentin and a simple model approach. *Eur. J. dentistry*. **3**, 32–41 (2009). PMC2647957 19262729

[52] Van Meerbeek, B. et al. Adhesion to enamel and dentin: Current status and future challenges. *Oper. Dent.* **28**, 215–235 (2003). 12760693

[53] Cruz, J. et al. Dentin permeability and nanoleakage of universal adhesives in Etch-and-rinse versus self-Etch modes. *Oper. Dent.* **46**, 293–305. 10.2341/19-276-l (2021). 34424991 10.2341/19-276-L

[54] Bourgi, R. et al. A literature review of adhesive systems in dentistry: Key components and their clinical applications. *Appl. Sci.* **14**, 8111 (2024).

[55] Mazzoni, A. et al. Effects of etch-and-rinse and self-etch adhesives on dentin MMP-2 and MMP-9. *J. Dent. Res.* **92**, 82–86. 10.1177/0022034512467034 (2013). 23128110 10.1177/0022034512467034 PMC3521453

[56] Kharouf, N. et al. Does adhesive layer thickness and tag length influence short/long-term bond strength of universal adhesive systems? An in-vitro study. *Appl. Sci.* **11**, 2635 (2021).

[57] Russell, R. G. G. J. B. Bisphosphonates: The first 40 years. *Bone* **49**, 2–19 (2011). 21555003 10.1016/j.bone.2011.04.022

[58] Yoshida, Y. et al. Comparative study on adhesive performance of functional monomers. *J. Dent. Res.* **83**, 454–458 (2004). 15153451 10.1177/154405910408300604

[59] da Silva, R. A. A., Trinca, R. B., Vilela, H. S. & Braga, R. R. Composite containing calcium phosphate particles functionalized with 10-MDP. *J. Dent. Res.* **103**, 427–433. 10.1177/00220345231225459 (2024). 38284313 10.1177/00220345231225459

